# Triglyceride-glucose index and overactive bladder syndrome: evidence from the NHANES 2005-2018

**DOI:** 10.3389/fendo.2025.1610140

**Published:** 2025-06-04

**Authors:** Baitong Chen, Jiongming Wang, Yueting Huang, Nanhui Chen

**Affiliations:** ^1^ The First School of Clinical Medicine, Guangdong Medical University, Zhanjiang, China; ^2^ Meizhou School of Clinical Medicine, Guangdong Medical University(Meizhou People’s Hospital), Meizhou, China; ^3^ Department of Urology, Meizhou People’s Hospital (Meizhou Academy of Medical Sciences), Meizhou, China

**Keywords:** overactive bladder syndrome, OAB, TyG index, cross-sectional study, risk assessment, NHANES

## Abstract

**Introduction:**

The relationship between the triglyceride-glucose index (TyG) and overactive bladder (OAB) is unclear. The aim of this study was to investigate the possible association between the TyG index and OAB.

**Methods:**

National Health and Nutrition Examination Survey (NHANES) data from 2005–2018 were used. The association between TyG index and OAB was assessed using univariate and multivariate logistic regression models. In addition, restricted cubic spline curves were used to examine the dose-response relationship between TyG index and OAB risk. Subgroup analyses and interaction analyses were then performed to assess differences in associations between subgroups.

**Results:**

A total of 14,059 participants with 3325 patients with OAB were included. In the fully adjusted model, each unit increase in the TyG index was associated with a 1.18-fold increased risk of OAB (OR = 1.18; 95% CI: 1.11-1.26; p < 0.001) and a 1.32-fold increased risk of OAB for Q4 compared to Q1 at the quartile level (OR = 1.32, 95% CI: 1.17- 1.50, p < 0.001), RCS analysis showed a positive linear association between TyG index and OAB, and subgroup analysis showed that the association between TyG and OAB was more pronounced in individuals younger than 60 years and in women (p for interaction < 0.05).

**Conclusion:**

The results of this study suggest that TyG index is positively associated with OAB, and this association was more pronounced in younger age groups and in females.TyG index, as a simple and cost-effective metabolic marker, may provide a potential tool for early screening of OAB, especially in individuals with metabolic abnormalities that have not progressed to significant metabolic diseases.

## 1 Background

Overactive Bladder (OAB) is defined by the International Continence Society as a chronic condition with urinary urgency as the main symptom, accompanied by urinary frequency, nocturia and urge incontinence (UUI), excluding clear pathological changes such as urinary tract infection. Epidemiological data show that the prevalence of OAB has been increasing over the past 20 years, with a global prevalence of OAB ranging from approximately 10.7% to 23.9%, and that its incidence increases significantly with age, especially in women over 44 years of age and men over 64 years of age (1). As a urological condition that severely affects quality of life (2), the medical costs of patients with OAB are more than two and a half times higher than those of patients without the condition (3), resulting in a significant burden on their families and social services. As a disease that seriously affects quality of life, the medical costs of OAB patients are more than 2.5 times higher than those of non-OAB patients, placing a heavy burden on their families and social welfare, and has become a major public health problem (4).

The etiology of OAB is thought to be multifactorial, involving neurogenic, myogenic, uroepithelial, or a combination of factors (5). Despite extensive research, the exact pathogenesis of OAB remains poorly understood, and it is possible that chronic inflammation (6) and elevated levels of oxidative stress may play a role in its development (7). Previous studies have shown that components of the metabolic syndrome (MetS), such as obesity, as well as diabetes mellitus, are associated with higher levels of inflammation and oxidative stress and are strongly correlated with OAB (8). IR may cause autonomic dysfunction, suggesting that insulin resistance (IR) may play a key role. IR may cause autonomic dysfunction and may be associated with OAB. Previous studies have shown that components of the metabolic syndrome (MetS), such as obesity and diabetes mellitus, have higher levels of inflammation and oxidative stress and are strongly correlated with OAB, suggesting that insulin resistance (IR) may play a key role. IR may cause autonomic dysfunction, thereby increasing bladder sympathetic tone, and hyperinsulinemia may directly stimulate urethral myocyte proliferation, which together contribute to the development of OAB (9).

Plasma insulin is the main indicator for assessing IR, and insulin levels are affected by a variety of factors, such as diet, exercise, and sleep (10), leading to greater fluctuations in test results, as well as disadvantages such as its high cost of testing and susceptibility to drug interference, and the results measured may not accurately reflect the patient’s IR level (11). The triglyceride-glucose index (TyG index) reflects the metabolic triglyceride and glucose metabolism, and these changes are closely related to IR, which is another new index used to assess IR in recent years. In comparison with the HOMA-IR model, the TyG index is more sensitive for the prediction and diagnosis of disease in the assessment of homeostatic models of IR and is more convenient and accessible in clinical practice (12, 13). Previous studies have shown that an elevated TyG index is strongly associated with a variety of conditions including asthma (14), kidney stones (15) and erectile dysfunction (16) and has demonstrated predictive value for cardiovascular events, atherosclerosis and chronic kidney disease in several large cohort studies (17).Although the association of TyG index with metabolic diseases has been confirmed by extensive studies, there is a gap in direct research on its association with OAB. Based on the above evidence, we explored the association between TyG index and OAB using data from the 2005–2018 U.S. National Health and Nutrition Examination Survey (NHANES), and we hypothesized that a higher TyG index is associated with an increased likelihood of OAB.

## 2 Materials and methods

### 2.1 Participant inclusion

NHANES is conducted by the National Center for Health Statistics and is a continuous, stratified, multistage sample survey. It integrates structured interviews and physical examinations to provide a comprehensive assessment of the health and nutritional status of groups of adults and children in the United States. Sampling strategies and detailed information used in this study were obtained from the NHANES website. All NHANES data are publicly available at https://www.cdc.gov/nchs/nhanes/ and written consent for the study was obtained from all participants.

**Figure 1:**
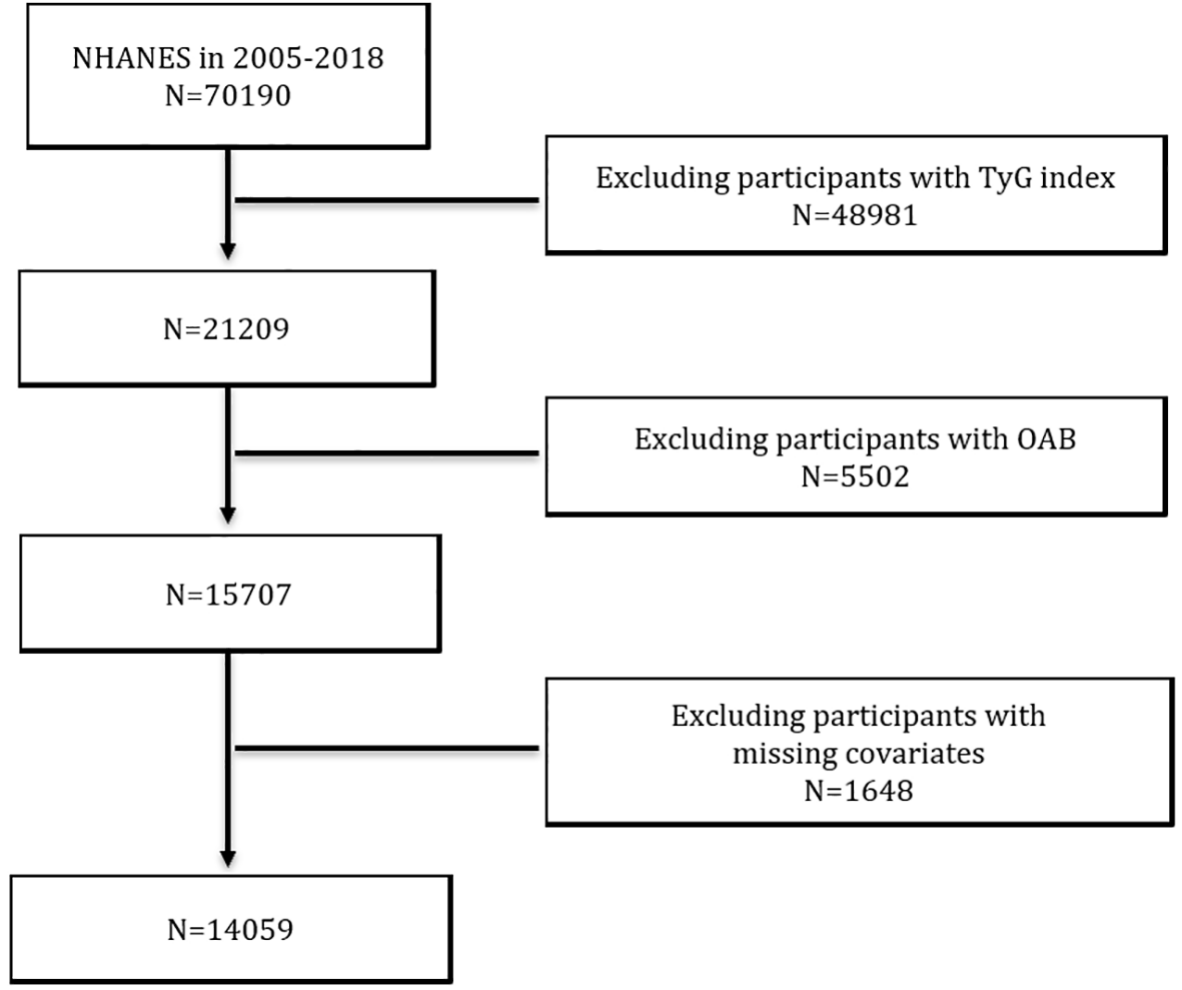
Flowchart of Participant Selection form NHANES Data (2005-2018).

In this cross-sectional study, we used data from seven consecutive NHANES cycles from 2005 to 2018. A total of 70,190 participants were included in these seven cycles. We excluded participants with missing data on the TyG index (n = 48981), participants with incomplete data on OAB-related questionnaires (n = 5502), and participants lacking information on covariates (n = 1648). Ultimately, this study identified 14,059 participants who met the inclusion criteria (**Figure 1**).

### 2.2 OAB diagnostic criteria and TyG index

Overactive Bladder Syndrome (OAB) is defined as an overactive voiding reflex that manifests itself in urge urinary incontinence (UUI) and nocturnal urinary frequency. We quantified overactive bladder syndrome using the Overactive Bladder Symptom Score (OABSS) questionnaire, and the diagnostic criteria for OAB were based on existing reports in the literature (18). The data collected here were obtained from questionnaires filled out by trained researchers during face-to-face interviews, and participants with a total score of 3 or higher were diagnosed with OAB. The NHANES questionnaire data and the OABSS score of symptoms were compared with the OABSS score. Frequency conversion criteria are shown in (**Table 1**) as in previous studies (6, 19).

**Table 1:** Conversion standards for symptom frequency recorded in the NHANES database and OABSS scores.

Based on NHANES Scores	Based on OABSS Scores
Frequency of Urgency Incontinence	Urgency Incontinence Score
Never	0
Less than once a month	1
Several times a month	1
Several times a week	2
Daily or nightly	3
Frequency of Nocturia	Nocturia Frequency Score
0	0
1	1
2	2
3	3
4	3
5 times or more	3

NHANES, National Health and Nutrition Examination Survey; OABSS, Overactive Bladder Symptom Score.

TyG index was calculated by the formula Ln [fasting TG (mg/dL) × fasting glucose (mg/dL)/2].17 Triglyceride and fasting glucose measurements were determined enzymatically on a Roche Modular P and Roche Cobas 6000 chemistry analyzer, respectively. Participants were categorized into 4 groups (Q1, Q2, Q3, Q4) based on the quartiles of TyG index, with group Q1 as the reference group.

### 2.3 Covariates

Based on the available literature (19), we assessed the potential impact of factors that may influence outcomes, considering variables identified as potential confounders. These factors included age (years), sex, race (non-Hispanic white or other), marital status (married/cohabiting, other), body mass index (BMI) in three groups: normal < 25.0, overweight 25.0-29.9, and obese ≥ 30.0), poverty income ratio (PRI), sleep hours, alcohol and smoking status, hypertension, diabetes, asthma, arthritis, and cancer.

### 2.4 Statistical analysis

Sampling weights were applied in all statistical analyses, which were performed according to the guidelines of the Centers for Disease Control and Prevention (CDC), and continuous variables were presented as means and standard deviations, and categorical variables were presented as percentages. We compared the baseline characteristics of non-OAB individuals with those of OAB individuals using independent samples t-tests, chi-square tests, and Mann-Whitney U tests. Logistic regression models were used to explore the independent relationship between the TyG index and OAB by dividing the TyG index into Q1-Q4 according to continuous variables and according to quartiles. There were three models, with no covariate adjustment in the unadjusted model; model 1 was adjusted for gender, age, race, BMI, alcohol consumption, and smoking; and model 2 was adjusted for gender, age, race, BMI, alcohol consumption, smoking, marital status, PRI, hours of sleep, hypertension, diabetes, asthma, arthritis, and cancer. In addition, we used restricted cubic spline curves (RCS) to explore the dose-response relationship between TyG index and OAB risk. Subsequently, we performed subgroup analyses stratified by age, PRI, race, sex, hypertension, diabetes mellitus, asthma, arthritis, and malignancy, and implemented interaction analyses to assess differences in associations between subgroups. All statistical analyses in this study were performed using R software (R 4.2.3) for data compilation and analysis. A two-sided p < 0.05 was considered statistically significant.

## 3 Results

### 3.1 Baseline characteristics of participants

A total of 14,059 participants were enrolled in this study; 10,734 were non-OAB patients and 3,325 were diagnosed with OAB (**Table 2**). Compared with participants in the non-OAB group, the prevalence of OAB was higher in the subgroups of ≥60 years of age, female patients, BMI ≥30, other races, PIR ≤1.30, alcohol consumption, and sleep duration ≤6 hours. The prevalence of OAB among nonsmokers was 49.8% (N = 1,655) compared with 50.2% (N = 1,670) among smokers, suggesting that smoking may have a smaller effect on OAB risk (P < 0.001). In addition, subgroup variables such as hypertension, diabetes, asthma, arthritis, cancer, and TyG index showed significant differences between the non-OAB and OAB groups (P < 0.001).

**Table 2:** Baseline characteristics of the study population. (weighted).

Variables	Non-OAB (N=10,734)	OAB (N=3325)	Total (N=14,059)	p-value
Age (%)				
20-39	4197 (39.1%)	478 (14.4%)	4675 (33.3%)	<0.001
40-59	3661 (34.1%)	957 (28.8%)	4618 (32.8%)	
≥60	2876 (26.8%)	1890 (56.8%)	4766 (33.9%)	
gender (%)				
Female	5067 (47.2%)	2031 (61.1%)	7098 (50.5%)	<0.001
Male	5667 (52.8%)	1294 (38.9%)	6961 (49.5%)	
BMI (%)				
<25	3387 (31.6%)	683 (20.5%)	4070 (28.9%)	<0.001
25-29.9	3678 (34.3%)	998 (30%)	4676 (33.3%)	
≥30	3669 (34.2%)	1644 (49.4%)	5313 (37.8%)	
Race (%)				
Non-Hispanic White	4941 (46%)	1463 (44%)	6404 (45.6%)	0.042
Other Race	5793 (54%)	1862 (56%)	7655 (54.4%)	
PRI (%)				
≤ 1.30	3096 (28.8%)	1237 (37.2%)	4333 (30.8%)	<0.001
1.31–3.50	4068 (37.9%)	1348 (40.5%)	5416 (38.5%)	
>3.50	3570 (33.3%)	740 (22.3%)	4310 (30.7%)	
Smoking status				
NO	5916 (55.1%)	1655 (49.8%)	7571 (53.9%)	<0.001
YES	4818 (44.9%)	1670 (50.2%)	6488 (46.1%)	
Alcohol Intake (%)				
No	3131 (29.2%)	1318 (39.6%)	4449 (31.6%)	<0.001
Yes	7603 (70.8%)	2007 (60.4%)	9610 (68.4%)	
Sleep Duration (%)				
7-9	6801 (63.4%)	1883 (56.6%)	8684 (61.8%)	<0.001
≤ 6	3501 (32.6%)	1183 (35.6%)	4684 (33.3%)	
> 9	432 (4%)	259 (7.8%)	691 (4.9%)	
Hypertension (%)				
No	7420 (69.1%)	1475 (44.4%)	8895 (63.3%)	<0.001
Yes	3314 (30.9%)	1850 (55.6%)	5164 (36.7%)	
Diabetes (%)				
No	9735 (90.7%)	2502 (75.2%)	12237 (87%)	<0.001
Yes	999 (9.3%)	823 (24.8%)	1822 (13%)	
Asthma (%)				
No	9290 (86.5%)	2715 (81.7%)	12005 (85.4%)	<0.001
Yes	1444 (13.5%)	610 (18.3%)	2054 (14.6%)	
Arthritis (%)				
No	8341 (77.7%)	1826 (54.9%)	10167 (72.3%)	<0.001
Yes	2393 (22.3%)	1499 (45.1%)	3892 (27.7%)	
Cancer (%)				
No	9917 (92.4%)	2819 (84.8%)	12736 (90.6%)	<0.001
Yes	817 (7.6%)	506 (15.2%)	1323 (9.4%)	
TyG index (%)				
Q1	2846 (26.5%)	641 (19.3%)	3487 (24.8%)	<0.001
Q2	2715 (25.3%)	791 (23.8%)	3506 (24.9%)	
Q3	2677 (24.9%)	863 (26%)	3540 (25.2%)	
Q4	2496 (23.3%)	1030 (31%)	3526 (25.1%)	

Values are, n (%) or mean (SE), TyG, triglyceride-glucose index; Q1, first quartile; Q2, second quartile; Q3, third quartile; Q4, fourth quartile;BMI, body mass index; PRI, Poverty income ratio.

### 3.2 Association between TyG index and OAB

Through one-way as well as multifactor logistic regression models, we observed a positive association between TyG index and OAB(**Table 3**). For the continuous TyG index variable, a positive association was demonstrated in the unadjusted model (OR = 1.39, 95% CI: 1.31-1.47; p<0.001), and this positive association remained stable in the partially adjusted model, Mode I, and in the fully adjusted model, Model II (OR = 1.18, 95% CI: 1.11-1.26; p<0.001), indicating that the risk of OAB would increase by 18% for each unit increase in TyG index. To further explore the gradient relationship between TyG index and OAB risk, we also converted TyG index from continuous to categorical variables (Q1, Q2, Q3, Q4) according to quartiles for sensitivity analysis and observed a significant positive correlation. In the fully adjusted model Model II, participants in Q2, Q3, and Q4 were associated with an increased risk of OAB compared to Q1.Q2 (OR = 1.15, 95% CI: 1.01-1.30, P = 0.029); Q3 (OR = 1.16, 95% CI: 1.02-1.31, P = 0.022); Q4 (OR = 1.32, 95% CI: 1.17-1.50, P < 0.001). The TyG index was significantly and positively associated with the risk of OAB, and this association remained robust after adjustment for socio-behavioral, economic, and comorbid factors, suggesting that the TyG index may serve as a potential metabolic marker for OAB.

**Table 3:** Multivariate logistic regression of the association of TyG index with OAB. (weighted).

Exposure	Nonadjusted Model	p-Value	Model I	p-Value	Model II	p-Value
	OR (95% CI)		OR (95% CI)		OR (95% CI)	
TyG index	1.39 (1.31-1.47)	p<0.001***	1.30 (1.22-1.38)	p<0.001***	1.18 (1.11-1.26)	p<0.001***
TyG index quartile						
Q1	Reference	–	Reference	–	Reference	–
Q2	1.29 (1.15-1.45)	p<0.001***	1.26 (1.11-1.42)	p<0.001***	1.15 (1.01-1.30)	p=0.029*
Q3	1.43 (1.28-1.61)	p<0.001***	1.31 (1.16-1.48)	p<0.001***	1.16 (1.02-1.31)	p=0.022*
Q4	1.83 (1.64-2.05)	p<0.001***	1.59 (1.41-1.79)	p<0.001***	1.32 (1.17-1.50)	p<0.001***

OR, odds ratio; CI, confidence interval; TyG, triglyceride-glucose index; Q1, first quartile; Q2, second quartile; Q3, third quartile; Q4, fourth quartile. * p-value < 0.001, ** p-value < 0.01, *** p-value < 0.001, * p-value < 0.05.

Model I: adjusted for gender, age, race, BMI, alcohol consumption, and smoking.

Model II: adjusted for gender, age, race, BMI, alcohol consumption, smoking, marital status, PRI, sleep duration, hypertension, diabetes, asthma, arthritis and cancer.

### 3.3 Restricted cubic spline curve

**Figure 2:**
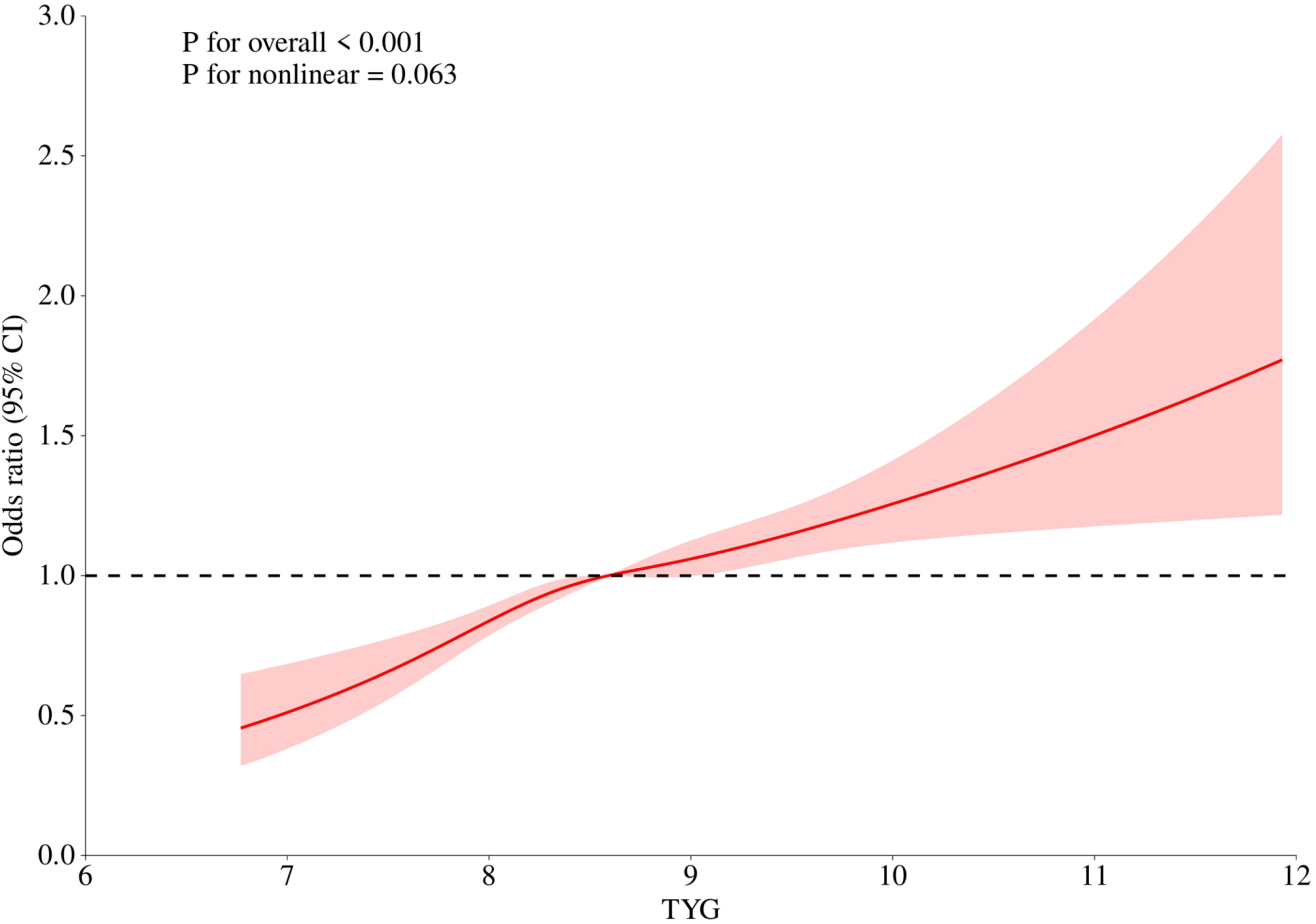
Linear relationship between triglyceride-glucose (TyG) index and OAB by the generalized additive model.

We explored the dose-response relationship between TyG index and OAB risk by using restricted cubic spline curves(**Figure 2**). After adjusting for relevant covariates in model II, there was a linear positive correlation between TyG index and OAB (P for total <0.001; P for nonlinear=0.063). The finding of this linear relationship provides an important quantitative basis for the role of the TyG index in the pathogenesis of OAB.

### 3.4 Subgroup analysis

**Figure 3:**
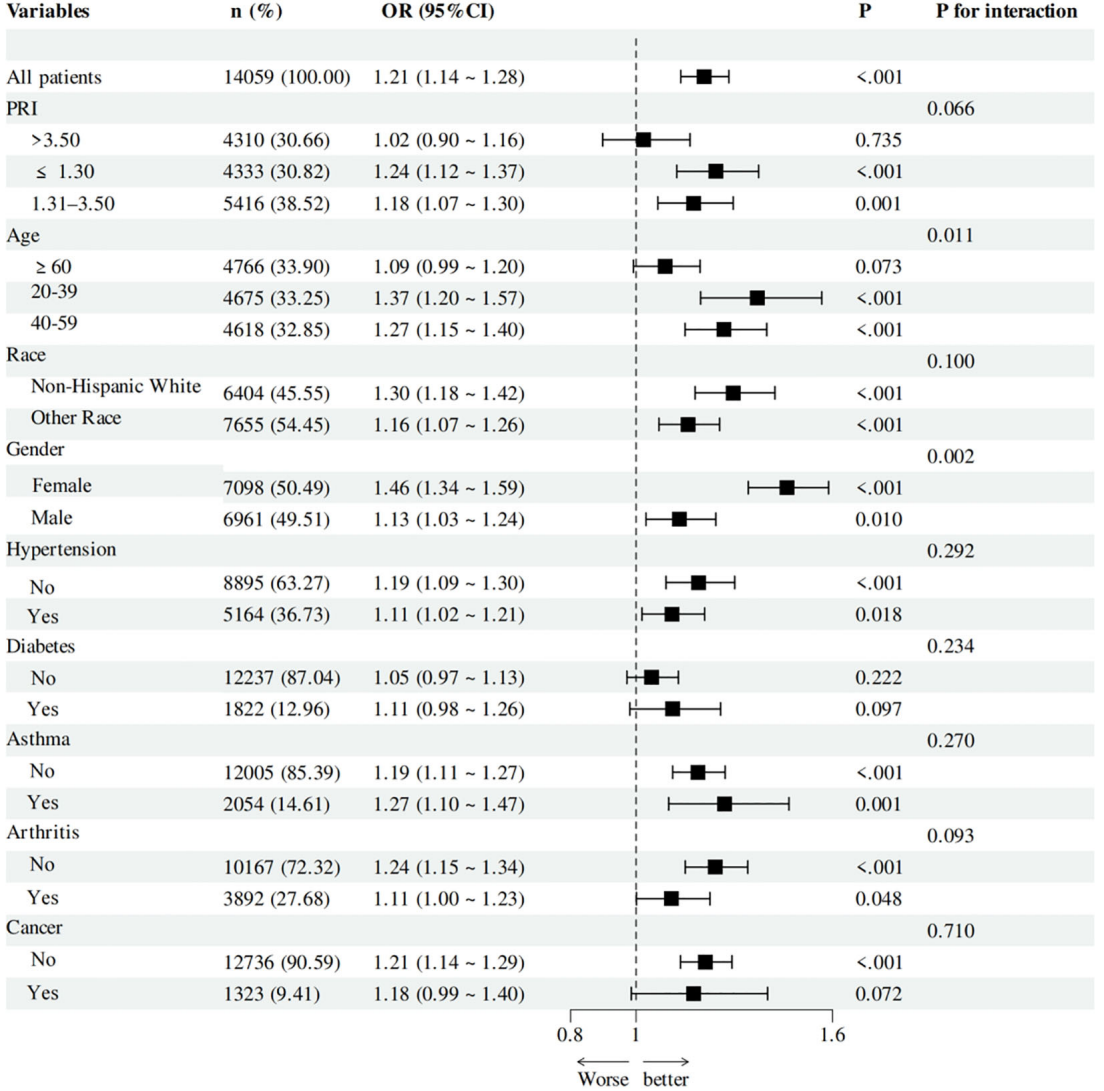
Subgroup analysis of TyG index associated with OAB (weighted).

To further elucidate the association between TyG index and OAB, we performed subgroup analyses within the PRI, age, race, gender, hypertension, diabetes, asthma, arthritis, and cancer subgroups (**Figure 3**). The results showed that the association between TyG index and OAB varied by age and gender status, and this association was more pronounced in individuals younger than 60 years (P for interaction = 0.011) and in women (P for interaction = 0.002). In contrast, this association did not reach statistical significance in individuals aged 60 years and older (P = 0.073). Furthermore, no additional interactions were observed for the association between TyG index and OAB in other subgroups (P for interaction >0.05).

## 4 Discussion

In this study, we explored the association between TyG index and OAB for the first time in a large population based on NHANES data. Through multidimensional analysis, we revealed an independent positive association between TyG index as a metabolic marker and the risk of OAB, and the results remained consistent after different stratification and sensitivity analyses.

In the fully adjusted model, there was an 18% increase in risk of OAB for each 1-unit increase in TyG index, and a significant dose-response relationship was observed when grouped into quartiles. The mechanisms underlying the relationship between TyG index and OAB may be independent of traditional metabolic syndrome components and may affect bladder function either directly or through other unmeasured pathways such as microangiopathy. Possible mechanisms include: Elevated TyG index is positively correlated with inflammatory markers such as interleukin-6 (IL-6) and C-reactive protein (CRP), which may contribute to the activation of related pathways to increase afferent neural sensitivity to the bladder (6, 16). At the same time, insulin resistance may lead to mitochondrial dysfunction and urethral (20) muscle fibrosis through oxidative stress injury mechanisms (17). Finally, through an autonomic dysregulation (21) mechanism, a high TyG index may increase sympathetic activity and induce detrusor overactivity (18). Together, these mechanisms contribute to OAB.

Subgroup analysis showed that the association between TyG index and OAB was still robust after adjustment for metabolic comorbidities such as diabetes mellitus and asthma, suggesting that the association between TyG index and OAB was more pronounced in people younger than 60 years and in female patients. Possible mechanisms include: a greater metabolic compensatory capacity in the younger population (22), and the associated inflammatory response due to insulin resistance is more likely to induce early bladder dysfunction (23). Females have higher levels of estrogen than males, and estrogen may enhance sensory afferents to the bladder by regulating TRPV1 channel expression (24). The urothelium of females is more sensitive to metabolic disturbances (8). The TyG index is independently and positively correlated with the risk of OAB, which is more robust in the younger population and in females. The TyG index, as a simple and inexpensive metabolic marker, may be a potential tool for early screening for OAB, especially in individuals with metabolic abnormalities who do not develop significant metabolic disorders. Prioritizing lifestyle interventions to reduce TyG index levels in patients with a high TyG index may be helpful in improving OAB symptoms. However, the clinical application of the TyG index needs to be further validated and its clinical value as a screening tool needs to be supported by further studies.

The present study also has several limitations. First, this study was based on a cross-sectional design, which provided evidence of a positive correlation between the TyG index and OAB. However, a causal relationship between these two variables could not be explained because OAB is a collection of symptoms caused by multiple factors. Although we controlled for some relevant confounders, we could not completely eliminate the effects of other confounding variables. Therefore, our results should be treated with caution. Second, the study data were derived from the US NHANES database and are not representative of the characteristics of the global population. In addition, the diagnosis of OAB is based on questionnaires, which may be subject to recall bias. Therefore, future studies need to validate the findings of this study in different populations and conduct multicenter prospective cohort studies to further clarify the causal relationship between TyG index and OAB.

## 5 Conclusion

This study demonstrates for the first time that TyG index is independently and positively associated with the risk of OAB, and that it is more pronounced in the younger age group and in females.TyG index may provide a potential tool for early screening for OAB, especially in individuals with metabolic abnormalities but who have not developed significant metabolic disorders. Further cohort studies are still needed in the future to demonstrate the potential association.

## Data Availability

The datasets presented in this study can be found in online repositories. The names of the repository/repositories and accession number(s) can be found below: https://www.cdc.gov/nchs/nhanes/.
